# Corrosion prevention of commercial alloys by air-water interface grown, edge on oriented, ultrathin squaraine film

**DOI:** 10.1038/s41598-019-50092-5

**Published:** 2019-09-17

**Authors:** Rajiv Kumar Pandey, Richa Mishra, Gopal Ji, Rajiv Prakash

**Affiliations:** School of Materials science and Technology, Indian Institute of Technology (BHU), Varanasi, Uttar Pradesh 221005 India

**Keywords:** Engineering, Materials science

## Abstract

Copper is one of the most demanded commercial metal/alloys in world market. The demand for copper in industries such as electrical, electronics, automobile, telecommunications, defence, etc. as well as in daily life has escalated in the recent years due to its versatile physical and chemical properties. However destruction of copper surface by any means, preferably corrosion, can limit its vast application. For protection from corrosion, various techniques are used to coat metal substrates with passivating materials. These techniques are either complex as well as expensive, or provide incomplete protection in acid media. To address these issues, floating film transfer method (FFTM) is utilized in this work for obtaining ultrathin film of squaraine (passivating molecule) as well as their easy and fast transfer over copper substrate. The squaraine film is deposited on copper substrate in layers, viz., 1 to 4 layers. The corrosion behavior is examined in 0.1 M HCl using electrochemical techniques as well as surface characterization techniques, which portray that copper corrosion is hampered in harmony with the layers deposited. Nearly 40% corrosion protection is reached for copper coated with 1 layer of squaraine. However, the protection is amplified up to 98% with 4 layers of squaraine, which clearly substantiates the supremacy of this coating method over reported methods of protection. This technique and the material (squaraine) are both for the first time being used in the field of corrosion protection. The easy growth of ultrathin film at air-water interface as well as its rapid transfer over substrate promotes use of FFTM for efficient corrosion protection on industrial scale.

## Introduction

Copper is a vital metal that is broadly used as a raw material in electronic, automobile, marine and domestic applications, such as, house wiring, ship hulls, under sea telephone cable, hydraulic tubing and propellers, because of its great bio-fouling resistance, corrosion resistance, mechanical strength and great electrical property^1,2^. However, the important functional properties of copper deteriorate faster in solutions having high amount of chloride ions^3,4^. Hence, it is necessary to increase the shielding strength of copper against the attack of chloride ions. There are several ways of protecting copper from its corrosion in aqueous solution; however, the use of organic coatings is most widely acceptable and efficient method for the job. The film of organic materials covers the surface of copper substrate and prevents it from being corroded by creating a physical barrier for aqueous solutions. However, the effectiveness of surface coating towards protection depends on the quality of film (i.e. degree of compactness) that in turn, is highly governed by the method adopted for film formation. Hence, it is necessary to select appropriate methods of coating for high quality corrosion prevention.

A large number of technologies have been used for film formation on the substrate such as, drop casting, spin casting, inkjet printing, etc.^5^. These techniques though easy to operate, but have major drawbacks of small area applicability and poor film quality. The reason of poor quality can be quoted to poor cohesive interactions among the molecules due to their random orientation during the film formation. Another issue with such film forming techniques is the non-uniform thickness of the film, which does not allow strong adherence of the film onto the substrate and causes disintegration of the film at several spots. Hence, it is necessary to produce thin and compact film over the substrate. There are technologies such as, Langmuir Blodgett (LB) and Langmuir Schaefer (LS) that employ air-water interface for large area, uniform thin film formation with controlled orientation of the molecules^6–9^. However, LB and LS techniques involve complex instrumentation and hence lack behind for large scale commercial use. Chemical vapor deposition (CVD) is also a good technique for thin film deposition^10^. However, CVD film is associated with the problem of small pores that can act as active centers for corrosion reactions. This problem is smartly dealt by atomic layer deposition (ALD) technique that produces high quality compact film over the substrate^10^. However, both ALD and CVD require complex instrumentation and skilled person. Hence, large scale use of these techniques in corrosion area is not a cost effective solution of the problem.

**Figure 1:**
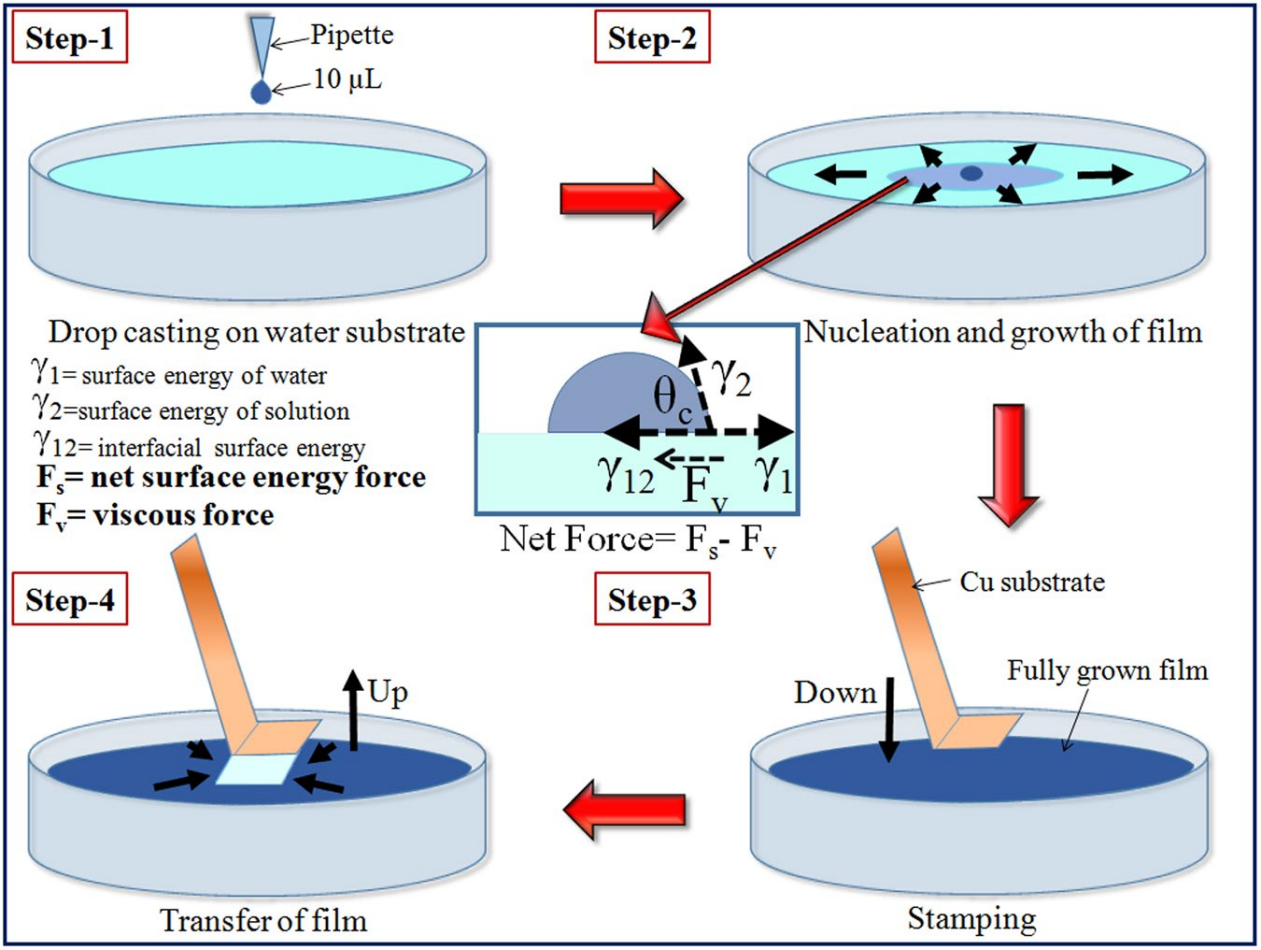
Schematic illustration of film formation by FFTM method and transfer of film over substrate.

To address above said issues, we are introducing Floating Film Transfer Method (FFTM) as a cost effective, high quality, large area and ultrathin film forming technique. This technique has close similarity with LS technique. The only technical difference is that LS technique requires application of pressure to the material’s solution for the film formation, whereas FFTM utilize inherent surface energy of the solutions as a driving force for film formation. FFTM requires two solvents for formation of ultrathin film at air-liquid interface: first, volatile and low surface energy (hydrophobic) solvent and second, non-volatile and high surface energy (hydrophilic) solvent. Organic material, which has to be deposited, is soaked in volatile solvent in optimized amount and a small drop (10–15 µL) of this solution is poured on hydrophilic surface. Due to surface energy difference, an ultrathin (~16 nm) film is formed at air-liquid interface (Fig. 1). The floating film is then easily transferred to the substrate by simple stamping like LS technique. Thus, it can be said that FFTM can be considered as a combination of drop cast and LS technique^11,12^. Details of this technique can be accessed in these reports^13–16^ and results section of this manuscript. One of the reports claims that FFTM can be used in large area applications, which makes this technique a strong contender in the coating industry^17^.

The aim of current work is to investigate the prevention of copper corrosion in chloride rich environment through copper substrates coated with squaraine (SQR) thin films formed by FFTM technique. As explained in above paragraph, FFTM is a cost effective technique producing high quality ultrathin films with large surface coverage which is being explored for the first time in corrosion prevention applications. SQR dye has a quadrupolar cyclobutadione (C_4_O_2_) central unit in its structure. The name ‘squarine’ to dye is given based on this square central unit. SQR belongs to the class of dyes that are widely known for their strong absorption and emission characteristics in longer wavelength region, wonderful stability under ambient conditions and very simple synthesis protocols^18,19^. Accordingly, it has been successfully implemented in gas sensors, bio-imaging probes, photodynamic therapy, nonlinear optics, organic solar cells and many other remarkable applications^20,21^. However, SQR has not been used for corrosion prevention of copper till date as per the best of our knowledge. SQR has very interesting chemical structure. It has hydrophobic functional group at one end and hydrophilic functional group at other end, which are connected to the square central unit in middle. This property renders its dissolution in both hydrophobic and hydrophilic solvent, which is good for producing films by FFTM. Metal surfaces are believed to be hydrophilic. This builds a possibility that hydrophilic portion of SQR can interact with surface, while other hydrophobic end will be in air. Thus, SQR modified metal surface will repel corrosive molecules and impart extra protection to metal. SQR contains nitrogen and oxygen lone pair of electrons that are shared with metal, resulting in strong bonding between SQR and metal. This is desirable for long term stability of films transferred onto metal surface. Further, these SQR coated metal surfaces have been checked for their corrosion prevention ability in 1 M HCl by single sine electrochemical impedance spectroscopy (SSEIS), Tafel polarization curves, differential pulse voltammetry (DPV), optical microscopy, HRSEM, EDAX, HRTEM, Uv-Vis spectroscopy, photo luminescence spectroscopy, FTIR spectroscopy, XRD and GIXRD techniques. The results show that few nanometers thick SQR film is highly efficient in prevention of copper loss in HCl, which strongly encourages industrial use of FFTM in corrosion prevention.

## Results

### Growth of film at air-water interface

**Figure 2:**
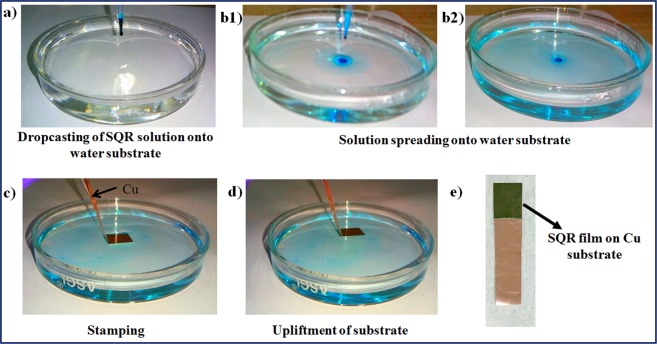
Real time images of film formation by FFTM method and transfer of film over Cu substrate.

It is very important here to discuss the growth mechanism of the film formed at air-water interface that will help in understanding the importance of particular orientation of molecules required for proper adhesion to the substrate. There are two solvents involved in this FFTM i.e. low surface energy solvent (LSES) and high surface energy solvent (HSES). The movement of LSES over HSES can be explained with ‘Marangonian flow’ concept^15^. A schematic presentation to describe this phenomenon is already shown in Fig. 1. A drop of LSES over HSES generates surface tension gradient at the interface of two immiscible liquids. This gradient promotes the spreading of LSES along the surface towards the regions of higher strain surface in centrifugal manner. Another important parameter simultaneously operating during spreading process is the viscosity of HSES. The viscosity of HSES opposes spreading of LSES over it. Hence, spreading of LSES is regulated by both surface tension gradient and viscosity of HSES as per equation: SS = F_D_ − F_V_. In this equation, SS is termed for spontaneous spreading of hydrophobic solvent, F_D_ is driving force that depends on the surface tension gradient and F_V_ is viscous force arising from viscosity of HSES. The driving force F_D_ can be further explained with a term spreading coefficient (S). This coefficient determines the possibility of spreading of one solvent over the other for film formation. Spreading coefficient is defined as S = γ_1_ − γ_2_ − γ_12_, where γ_1_ represent the surface energy of hydrophilic solvent, γ_2_ shows surface energy of the solution of material (to be deposited) in hydrophobic solvent and γ_12_ express the surface energy of the interface^15^. For spreading of the film, S must be greater than 0. Hence, both the solvents should be selected accordingly. It is evident from above discussion that hydrophilic solvent acts as substrate for deposition of the material’s film. In our case γ_1_ (water) is 72 mN m^−1^, γ_2_ (chloroform) is 27.5 mN m^−1^ and γ_12_ is 31.6 mN m^−1^ ^15^. Then, S is calculated as 12.9 mN m^−1^ (spreading speed 3.4 √Kg/s^2^), which is greater than zero and shows validity of solvent selection for film formation. Real time images of film formation by FFTM method and transfer of film over Cu substrate are shown in Fig. 2.

### Characterization of squaraine

**Figure 3:**
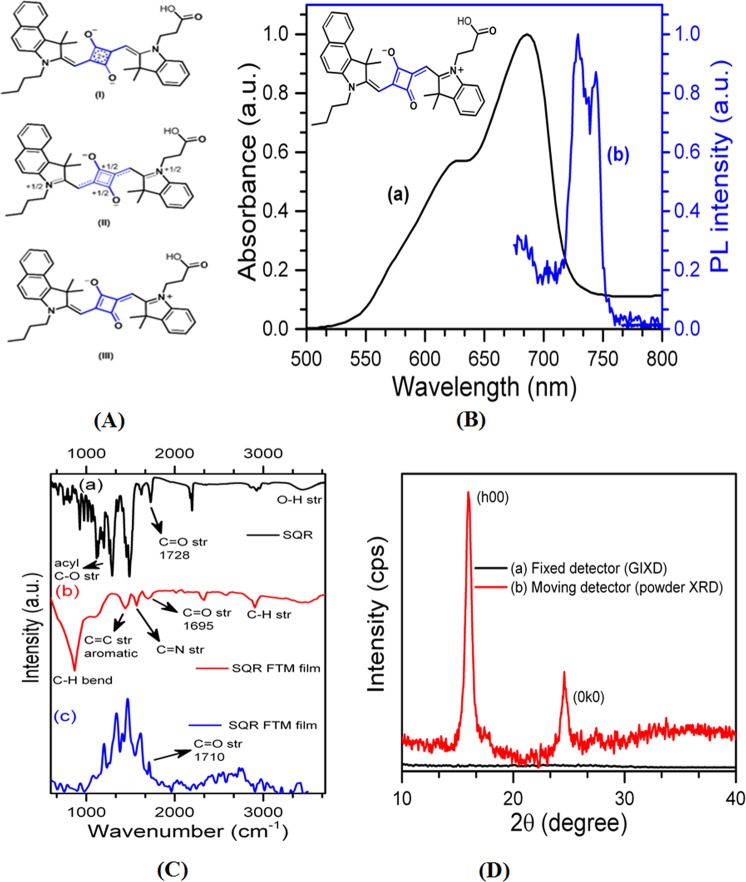
(**A**) Delocalization of the charge over entire SQR molecule and different forms (I, II and III). (**B**) UV-vis and PL spectra of SQR. Inset- chemical structure of SQR, (**C**) FT-IR of (a) free SQR, (b) SQR FTM film and (c) Raman spectra of SQR FTM film and (**D**) GIXRD spectra of SQR coated substrate as well as XRD spectra of SQR.

The most reliable characterization of SQR is the identification of the central unit of C_4_O_2_^18,19^. The central ring is basically an electron deficient ring and behaves as a donor-π-acceptor-π-donor system. The delocalization of the charge occurs over the entire molecule, which can be presented in different forms (I, II and III) as shown in Fig. 3A.

For basic characterization of SQR, one layer of SQR film was transferred on quartz substrate by FFTM and studied by UV-vis and PL spectroscopy (Fig. 3B). In UV-vis spectrum, a sharp absorption band appeared at 686 nm (λ_max_) in the long wavelength region, which exhibited π-π* (S_1_ − S_0_) transitions corresponding to the central ring (C_4_O_2_). A hypsochromic vibronic shoulder also appeared at 625 nm, which could indicate dye aggregation. The emission spectra of SQR showed two peaks at 728 and 743 nm, which projected that SQR was emitting in NIR frequency range^18–23^. Furthermore, functionality of SQR as well as its interaction with Cu substrate was validated by FTIR spectroscopy. Both SQR in powder form and as coated on Cu are examined and related FTIR spectra are presented in Fig. 3C. In the spectrum of SQR, the characteristic peak of saturated carboxylic acid (C=O stretching) appeared at 1728 cm^−1^. Whereas, C=O stretching peak shifted towards lower wave number (1695 cm^−1^) for SQR FTM film (Fig. 3C). This shift in C=O stretching peak clearly signified the interaction between Cu and SQR via hydrophilic carboxylic (C=O) end^24^. Furthermore, raman spectroscopy investigation of weak C=O peak at 1710 cm^−1^ ascertained the interaction between Cu substrate and hydrophilic carboxylic end of SQR dye. Due to polarizability variation during the vibrational mode, a weak but clear C=O band appeared in the spectrum^25^. Thus, UV-vis and FTIR analysis together confirmed that SQR was successfully transferred over Cu substrate and its hydrophilic C=O group was attached to the substrate.

To examine self-assembly and orientation in the SQR film, XRD spectrum was recorded in two modes as shown in Fig. 3D. First mode was powder XRD, where detector was fixed while source was moving with 2θ with respect to sample. The second mode was GIXD of pristine films with 0.2° grazing angle (equipped with in plane diffractometer and out of plane moving detector). GIXD pattern of SQR film did not show any peak. However, powder XRD displayed two intense peaks at 15.96° and 24.57°, which could be indexed as (h00) and (0k0) diffraction peak of SQR^23,26^. The interplanar spacing of (010) planes of SQR film was found to be 0.36 nm. Peak absence in GIXD of SQR film might be observed due to orientation (stacking) of molecules in a particular direction. Peak intensity investigation of pure SQR revealed well-grown and highly oriented domain rich films without thermal preparation as also complemented by HR-TEM and SAED images discussed below.

**Figure 4:**
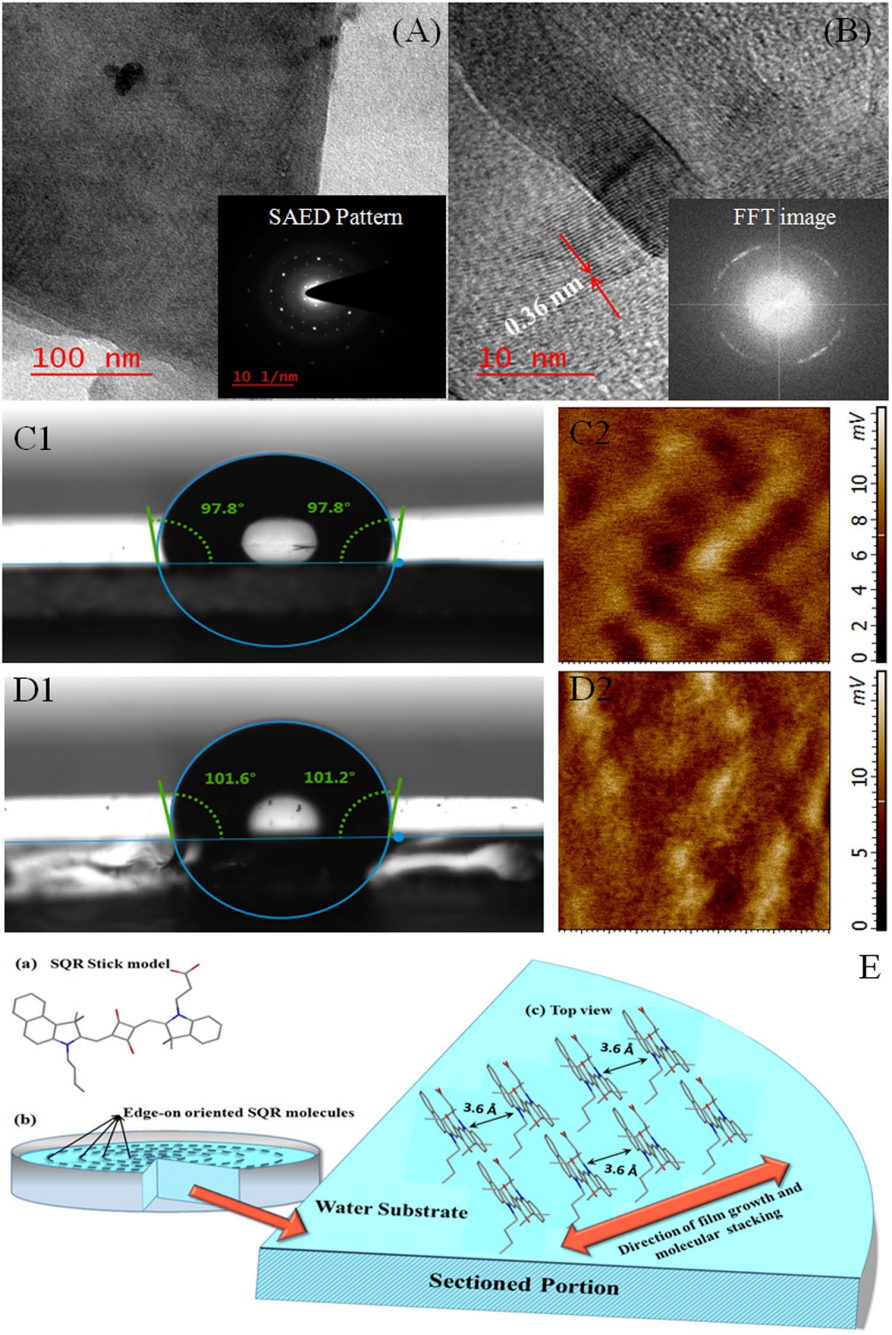
HRTEM images at (**A**) low magnification and (**B**) high magnification. Inset- (**A**) SAED pattern and (**B**) FFT of same region, surface energy/contact angle and KPFM measurements of SQR casted ITO substrate with (**C1**,**C2**) hydrophilic end upside (**D1**,**D2**) hydrophobic end upside and (**E**) schematic illustration of film growth over water substrate and molecular stacking (a) SQR stick model, (b) edge on stacking and (c) growth of SQR molecules.

HRTEM images and SAED pattern were recorded to examine the morphology, crystallinity and most important the orientation of as deposited film. It is noteworthy here that SQR film thickness of 10–20 nm and sub mm size can form free standing film^23^. Low magnification HRTEM image of free standing film (Fig. 4A) revealed that well large crystallites of SQR were formed. SAED pattern of the same region also authenticated crystalline nature of SQR film. Further, investigation of the same region of free standing SQR film via high magnification HRTEM (Fig. 4B) demonstrated that SQR was aligned along (0k0) plane, which notified π-π stacking of SQR. In order to show focus of the beam on sample area in HRTEM, Fast Fourier Transform (FFT) (Fig. 4B-inset) of same region was recorded which validated the formation of π-π stacked structure. Brinkmann *et al*.^27^ has already reported highly oriented crystalline films of other molecules with edge-on lamellar morphology, similar to as obtained in this case. The spacing between the lattice fringes was measured and found to be 0.36 nm, which was equivalent to d-spacing of (010) plane or π-π stacking distance of SQR backbones as discussed above in XRD results. This confirmed that π-π stacked lamellar structure of polymer backbones with edge-on arrangement was present in film over the substrate, which was highly desirable for adherence and protection of surface.

It is important to note that HR-TEM exhibited π-π stacking of the planes. However, powder XRD showed (h00) and (0k0) planes which highly confirms the edge-on orientation of SQR film. As per literature, organic molecules form a stable film with alkyl chain lying over high energy surface^23^. Therefore, SQR film will be energetically stable only with alkyl side chain lying over the water substrate. In this scenario COOH, O and N present in plane of molecule will interact with high energy surface (water substrate) during deposition via horizontal stamping and alkyl group will be exposed to the external ambient. Thus, alkyl group will minimize the energy imbalance. The edge-on orientation is highly desirable for planar surface protection due to the presence of COOH, O and N in plane of molecule.

Figure 4C,D present the surface contact angles and corresponding KPFM images of the SQR film lifted in two ways. Figure 4C1,C2 is for SQR film (type I) lifted by dipping the substrate first into the water subphase, then slowly lifting the floating SQR film from below the water subphase. Whereas Fig. 4D1,D2 show images for SQR film (type II) lifted via tapping of the substrate from upside over the water subphase. We are assuming that type ISQR film is hydrophilic (that adheres to the Cu substrate coated for all investigations) and type II film is comparatively hydrophobic in nature (also acts as surface of modified Cu substrate). Surface contact angle measurements clearly justified our assumption that type I film is hydrophilic and type II film is hydrophobic^23,28^. Although surface contact angle is greater than 90° for both kind of films, yet we can say that more hydrophilic part of the film adheres to the hydrophilic Cu substrate. This fact was also confirmed by KPFM images of SQR film. The scale of voltage is slightly higher for type II SQR film, which revealed that acid exposed surface of SQR modified Cu is hydrophobic again strengthening our assumption. Based on the above results, a schematic has been designed about SQR structure, film growth and its orientation & stacking in the film, and presented in Fig. 4E(a–c).

### Characterization of SQR coated copper substrates

SQR film was formed at air-water interface by FFTM and four layers (1, 2, 3 and 4) of SQR were transferred over Cu substrate. HRSEM images, EDAX mappings and differential pulse voltammetry (DPV) measurements were recorded to ensure the transfer of the film over Cu substrates. Figure S1 (Supporting Information) shows HRSEM images and corresponding EDAX mappings of polished Cu as well as SQR coated Cu substrates. HRSEM images did not find any remarkable difference in surface morphology between Cu and SQR coated Cu. However, EDAX mappings revealed that concentration of C and N increased with the number of layers transferred. This fact was also confirmed by EDAX spectra of the same regions (Table S1, Supporting Information). Thus, it was evident that the layers were successfully transferred over Cu substrates.

**Figure 5:**
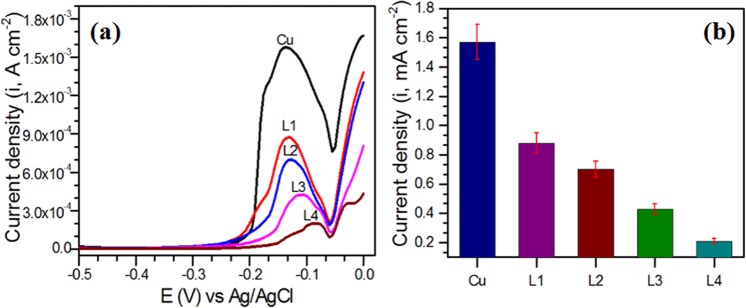
(**a**) DPV curves showing oxidation behavior of Cu substrates coated with different layers of SQR and (**b**) corresponding peak oxidation current.

To have preliminary idea about prevention ability of SQR, DPV analysis of the coated substrates were performed. The DPV curves for Cu and SQR coated Cu are presented in Fig. 5a,b. The oxidation current was lowered for SQR coated copper substrates. Also, the peak was shifted towards positive values with the number of layers. Both the facts indicated that SQR was successfully transferred over copper and prevented its dissolution in 0.1 M HCl^29^. The maximum reduction in current values (8 times) and shifting of oxidation peaks was achieved with 4 layers of SQR over copper. Besides, analysis of corroded copper and SQR coated copper were performed by HRSEM and EDAX techniques and shown in Fig. S3 (Supporting Information).The images portrayed that HCl attacked at several places on copper surface, which caused severe corrosion of copper and appeared as if it was broken at several places. In contrast, the surface morphology of the SQR coated copper substrates projected that corrosion of copper was seriously stopped; however, the prevention ability of 1 layer SQR coated Cu was the least and increased significantly with increase in the number of layers. EDAX mappings of copper substrates projected that corroded surfaces were covered with C, N and chloride. The maximum chloride concentration was found on corroded copper, while it was severely decreased for SQR coated Cu. Also, concentration of C and N was the highest for 4 layers SQR coated Cu and decreased with number of layers. These facts suggested that prevention of Cu loss (dissolution) in HCl was achieved in accordance with the layers transferred over Cu substrates.

## Discussion

**Figure 6:**
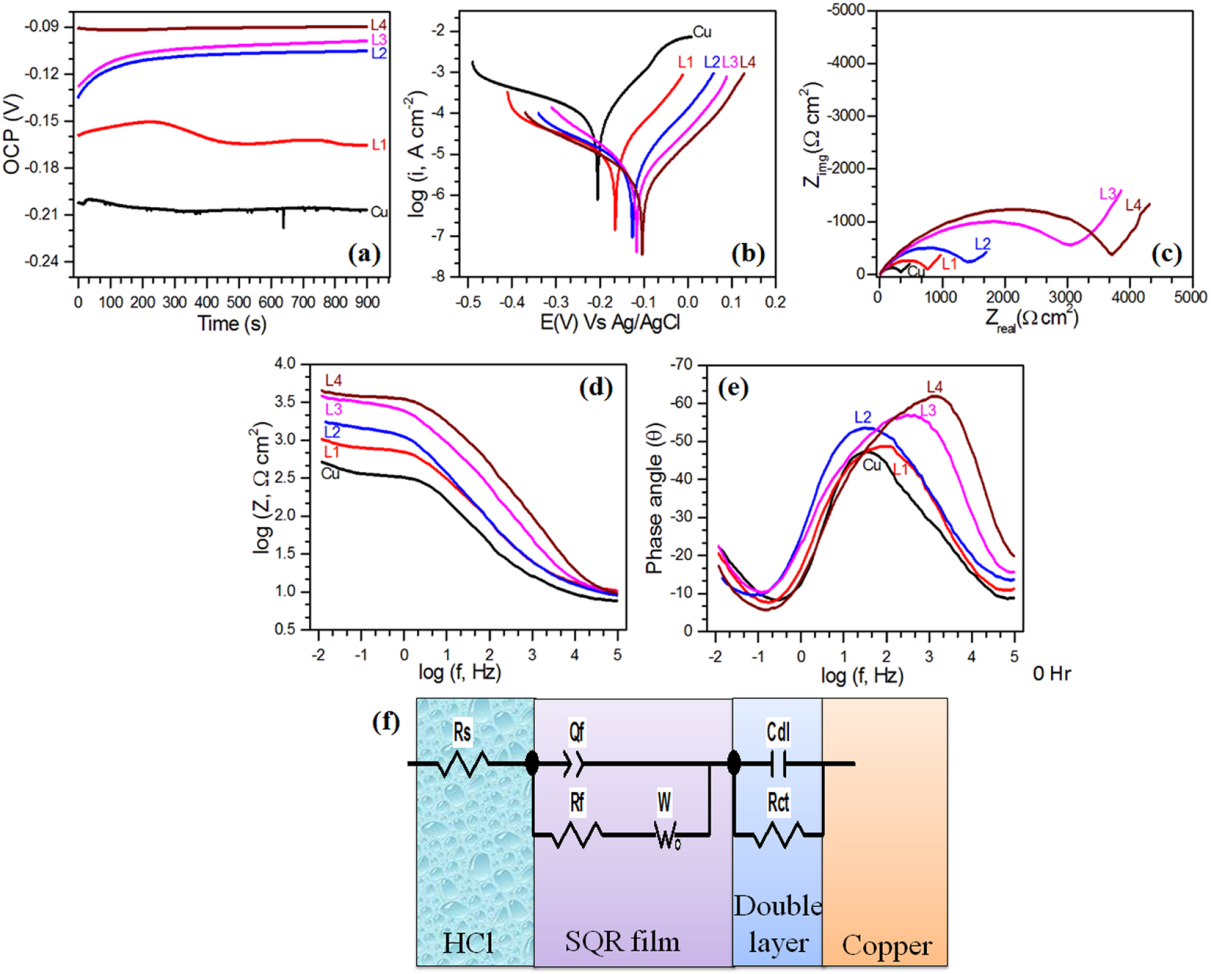
(**a**) OCP curves. (**b**) Tafel polarization curves of Cu and SQR coated Cu substrates after 15 min immersion in 0.1 M HCl at 299 ± 2 K. (**c**) Nyquist plot. (**d**) Bode modulus plots and (**e**) Bode phase plots of Cu and SQR coated Cu substrates after 15 min immersion in 0.1 M HCl at 299 ± 2 K. (**f**) Circuit model used for fitting the response curves.

Figure 6a,b presents open circuit potential (OCP) curves and Tafel curves for Cu as well as SQR coated copper substrates obtained in 0.1 M HCl. For Cu, OCP was slightly positive at 0 min in comparison to OCP at 15 min. This could predict that pristine Cu was having a layer of copper oxide on the surface, which was obviously expected. This layer could not withstand acidic environment (0.1 M HCl) after some time, as it got disrupted and thus could not protect Cu. OCP curve of 1L-SQR coated Cu suggested that SQR was preventing corrosion as the potential of Cu substrate shifted towards positive values vs. pristine Cu^30^. However, the shape of OCP curve further indicated that 1L-SQR could not cover the whole surface of Cu. OCP curves of 2L-SQR and 3L-SQR coated Cu substrates were found more positive than pristine Cu and 1L-SQR coated Cu, which suggested that surface coverage increased with the subsequent layering of SQR. Further, OCP curve for 4L-SQR coated Cu was almost constant for 15 min, with the highest positive value among all. This indicated that fourth layering of SQR over Cu covered its area to maximum thus preventing its corrosion with the best capacity. This explanation of prevention was done based on following equation^31^:

1 $$T=1-{\delta }^{N}$$

where *T* indicates density of uncovered points on the surface, *δ* relates with defects density and *N* is numbers of coated layers. This equation suggests that coverage of surface will subsequently increase with the number of layers transferred on the substrates, which supports our explanation. In any case, the potentials were showing more stability after 15 min in comparison to the beginning potential. Hence, Tafel and impedance experiments were performed only after 15 min of immersion of the substrates in 0.1 M HCl.

Figure 6b exhibits Tafel curves of pristine Cu and SQR coated Cu substrates, showing their polarization behavior. The curves exhibited shifting of potential (corresponding to the lowest current) towards more positive values for SQR coated Cu with reference to pristine Cu. Also, the lowest point of current density (log i) curve shows a decreasing trend with subsequent layers indicating decreasing value of current. Both these observations validated the prevention of Cu corrosion by SQR coating in 0.1 M HCl^29,30^. For better insight, Tafel curves were fitted by CHI-7041 C software (CH instruments, USA) and the exact parameters obtained are reflecting quantified changes in corrosion behavior of Cu. The parameters were: equilibrium corrosion potential, E_corr_; corrosion current density, I_corr_; anodic and cathodic curves slopes, b_a_ and b_c_; and polarization resistance, R_p_. The prevention efficiencies based on I_corr_ (µ_p_) and R_p_ (µ_Rp_) were determined according the following equations^32^:

2 $${\mu }_{p}( \% )=\frac{{I}_{{\rm{corr}}}^{0}-{I}_{corr}^{i}}{{I}_{{\rm{corr}}}^{0}}\times 100\,$$

3 $${\mu }_{Rp}( \% )=\frac{{R}_{{\rm{P}}}^{{\rm{i}}}-{R}_{P}^{0}}{{R}_{{\rm{P}}}^{{\rm{i}}}}\times 100$$

where superscripts ‘i’ and ‘0’are used for SQR coated Cu and pristine Cu in 0.1 M HCl, respectively.

Table 1 exhibits the changes in different parameters with the number of SQR layers transferred on Cu substrate. The E_corr_ of pristine Cu and 1L-SQR coated Cu were −190 mV and −161 mV respectively, which indicated that SQR was protecting Cu in HCl since E_corr_ was more positive^33^. With increase in the number of layers, E_corr_ started to move towards more positive potentials, which portrayed that thermodynamic stability of the system increased. The similar trend of shifts in potentials was also observed in OCP curves. The coating served as a physical barrier to H^+^ and Cl^−^ ions, that means reduction of H^+^ to H_2_ and oxidation of Cu to Cu^2+^ was prevented by SQR film. It was noticed that b_c_ was greater than b_a_; and b_c_ changed more than b_a_ with increase in the numbers of layers, which documented that conversion of H^+^ to H_2_ was more strongly prevented than oxidation of Cu to Cu^2+^ ^34,35^. Furthermore, prevention of corrosion reactions was directly significant from the I_corr_ values. I_corr_ value for pristine Cu was 86 µA cm^−2^, while it was 12 µA cm^−2^ for 1L-SQR coated Cu. Based on I_corr_ values, it was observed that efficiency of prevention was magnified with the number of layers as the openings on the Cu surface were reduced with multiple coatings (layers of the film). For 4L-SQR coated Cu, I_corr_ was just 2 µA cm^−2^ revealing only 2% corrosion of Cu in 0.1 M HCl. The prevention efficiency of SQR could also be checked by comparing R_p_ values. The polarization resistances of SQR coated Cu substrates were higher than pristine Cu, which suggested that equilibrium of the substrates could not be disturbed easily. This intended that SQR coated Cu system was more stable towards corrosion reactions (either cathodic or anodic) and these reactions could not be accelerated straightforwardly as it could be done in case of pristine Cu in HCl^36,37^.

**Table 1:** Corrosion parameters obtained from Tafel polarization curves of Cu and SQR coated Cu substrates after 15 min immersion in 0.1 M HCl at 299 ± 2 K.

Substrate	−E_corr_ (mV)	I_corr_ (µA cm^−2^)	b_a_ (mv dec^−1^)	−b_c_ (mv dec^−1^)	µ_p_ (%)	R_p_ (Ω cm^2^)	µ_Rp_ (%)
Cu_Blank	190	86	90	234	—	331	—
Cu_1L SQR	161	12	85	233	86	2247	85
Cu_2L SQR	119	5	89	183	94	5221	94
Cu_3L SQR	105	3	94	125	97	7184	95
Cu_4L SQR	091	2	93	166	98	11093	97

Figure 6c–e presents Nyquist, Bode modulus and Bode phase plots for pristine Cu as well as SQR coated Cu in 0.1 M HCl. Bode modulus plots reveals that impedance of SQR coated Cu substrates was higher than pristine Cu. The increase in quantity of impedance followed the number of layers and indicated that 4L-SQR coated Cu was giving the highest impedance. Bode phase plots showed that phase angle difference between applied voltage and current response was enhanced with the number of layers, which suggested that charge distribution on the surface was becoming more uniform. Both these observations advocated that corrosion of Cu was effectively prevented^38,39^. Furthermore, Nyquist plots of pristine Cu and SQR coated Cu could be divided in two segments: first, big semi-circular loop; and second, line/incomplete loop in low frequency zone. These observations suggested that corrosion of either pure Cu or SQR coated Cu was two time constant process, revealing that two interfaces were formed between electrolyte and Cu. This was also supported by bode phase angle plots. The size of response curves for SQR coated Cu was greater than for pure Cu, which specified that Cu corrosion in hydrochloric acid was prevented by SQR film^40,41^. Also, the increase in size followed the number of layers on Cu substrate, which clearly signified that corrosion was prevented in a better way with three or four layers of SQR over Cu.

To quantify the effects of SQR layers on Cu corrosion, impedance curves were fitted with a circuit model shown in Fig. 6f. As per the model, the corrosion behavior could be defined with following parameters: solution resistance, R_s_; constant phase capacitance of SQR film, Q_f_; resistance of SQR film/oxide film (in case of pristine Cu), R_f_; double layer capacitance of acid-cu interface, C_dl_; and charge transfer resistance of acid-Cu interface, R_ct_. The total resistance (impedance, Z) of the circuit can be described by the equation:

4 $$Z={R}_{s}+\frac{1}{{(jw)}^{\alpha }{Q}_{f}+\frac{1}{{R}_{f}+\frac{(1-j)W}{\surd \omega }}}+\frac{1}{j\omega {C}_{dl}+\frac{1}{{R}_{ct}}}$$

where j is √ − 1 and α denotes quality of charge distribution on the surface. In the equation, first term is for solution, second term is for SQR film and third term is for corrosion reactions at acid-Cu interface. All the experiments were performed in 0.1 M HCl; hence, HCl was test solution and corresponded to R_s_ in the circuit, as per conventional circuit fitting procedures. Second term was assigned to SQR film because it was the first interface formed between solution and Cu surface. Third term is assigned to acid-Cu interface because it was the second interface formed between solution and Cu surface. This interface could form due to insufficient coverage of Cu substrate by SQR film. So, solution could reach to base substrate through openings in the film. The χ^2^ values for the fitting were sufficiently low (10^−3^ order) and fitting errors in Z measurements were less than 5%, which indicated that corrosion of both pristine Cu and SQR coated Cu in 0.1 M HCl could be confidently explained with the proposed model. The prevention efficiency of SQR was determined based on R_ct_ values using the equation described below^42^:

5 $${\mu }_{{R}_{ct}}( \% )=\frac{{R}_{ct}^{i}-{R}_{ct}^{0}}{{R}_{ct}^{i}}\times 100$$

where superscript ‘i’ and ‘0’ are used to denote presence and absence of SQR film on Cu.

There were two zones on the Cu surface: first, covered with SQR; and second, openly accessible. Accordingly, electrolyte could reach to Cu substrate via micro pores of SQR film and openings of the surface. Data analysis of Table 2 disclosed that Q_f_ values decreased while R_f_ values raised with the number of SQR layers on Cu, which indicated that prevention ability of SQR increased with increase in number of layers^43,44^. The reason could be quoted as decrease in the porosity of SQR film with the number of layers, which was responsible for reduced acidic exposure of Cu beneath the layers. Due to increase in local density, the resistance of film subsequently increased with the number of layers. In the circuit model, W (modulus of Warburg impedance) is also used in series with R_f_. W is used to explain diffusion of electrolyte or diffusion of chloro-copper species through SQR/Cu corrosion products layering. The W values suggested that this reaction slowed down with the increase in number of layers. On the other side, corrosion reactions occurring at exposed surface could be analyzed through C_dl_ and R_ct_ values. The charge storage capacity of the acid-Cu interface (C_dl_) was significantly lowered with the number of layers on Cu substrate. This occurred as a result of decrease in number of surface openings, which actually indicated the higher surface coverage of Cu surface by SQR film. Accordingly, the charge transfer resistance also increased as the transport of charge across the acid-Cu interface was impeded by SQR film (barrier effect). Thus, it was observed that both R_f_ and R_ct_ participated to slow down the Cu corrosion in 0.1 M HCl. Hence, prevention efficiencies based on impedance analysis were calculated from total resistance against corrosion (R_f_ + R_ct_). The enhancement in prevention efficiency showed combined effect of decrease in porosity of SQR film and reduction in number of surface openings with the increase in number of layers. Based on all above results and discussion, a schematic is illustrated in Fig. S4 (Supporting Information) to show the corrosion inhibition activity of SQR.

**Table 2:** Corrosion parameters obtained after fitting of the response curves of Cu and SQR coated Cu substrates after 15 min immersion in 0.1 M HCl at 299 ± 2 K with circuit model.

Substrate	R_s_ (Ω cm^2^)	Q_f_ (µS.s^α^)	α	R_f_ (Ω cm^2^)	W (S.s^0.5^)	C_dl_ (µF cm^−2^)	Rct (Ω cm^2^)	µ_r_ (%)	χ^2^	Z_error_ (%)
Cu_Blank	7	900	0.529	136	0.0142	142	189	—	1.51 × 10^−3^	3.88
Cu_1L SQR	12	191	0.624	308	0.00862	170	198	36	2.31 × 10^−3^	4.80
Cu_2L SQR	8	316	0.582	959	0.00738	113	548	78	1.94 × 10^−3^	4.40
Cu_3L SQR	9	41	0.718	1353	0.00185	75	1319	88	1.27 × 10^−3^	3.56
Cu_4L SQR	8	16	0.753	1481	0.0024	23	1927	90	1.33 × 10^−3^	3.64

As a conclusion of the work, it can be said that this work focuses on the issues of thin and compact coating application for corrosion inhibition. A very new technique, never used before for corrosion prevention, FFTM is used to produce ultra-thin SQR film formed at air-water interface and transferred smoothly on Cu substrates. Thus coated Cu substrates were characterized with various techniques, and tested for prevention of corrosion by electrochemical and surface analysis techniques. UV-vis spectroscopy, PL spectroscopy and FT-IR spectroscopy confirmed that SQR used for the film formation was good in characteristics. Raman spectroscopy, HRTEM images, SAED pattern, live FFT image and XRD patterns revealed that SQR film formed at air-water interface was crystalline and highly ordered with long range π-π stacking. Coated Cu substrates were preliminary examined by optical microscopy, HRSEM, EDAX and DPV techniques, which clearly showed that SQR layers were successfully transferred over Cu substrates and having a great influence on corrosion prevention efficiency of Cu. Corrosion analysis was performed in detail by EIS and Tafel polarization techniques, which portrayed that corrosion of Cu in 0.1 M HCl was impeded in accordance with the layers transferred. The maximum corrosion prevention (90%, EIS and 98%, Tafel curves) was acknowledged for Cu coated with 4 layers of SQR. The reason was very clear, i.e., the porosity of film decreased with increase in number of layers. Thus, the analysis strongly recommends FFTM as a new method of coating the base (metal) substrates for prevention towards corrosion. Although we have used SQR in this work, we can use many other cheap materials coated via FFTM for corrosion prevention.

## Methods and Materials

### Materials and preparation of the copper substrate

Thin Cu sheet of 99.9% purity was purchased from the metal market in Varanasi (India) and used as substrate material for this work. SQR Dye, chloroform, double distilled water (resistivity = 18.0 MΩ) and HCl were purchased from Merck, India.

Preliminary preparation of Cu sheet was done by rubbing it with emery paper. This was performed in a sequence from low grade number to high grade number (1 to 5) emery paper of Sianor B, Switzerland. The sheet was rubbed in both parallel and perpendicular directions for 5 minutes with each number of emery papers. Then, the sheet was dip cleaned in 0.05 M HCl and immediately wiped with lint free tissue. As a final step, the sheet was abraded with emery paper of grade 6. Thus prepared sheet was cut into specimens of 6 × 1 cm^2^ and used as substrate for the study. For electrochemical investigation, only 1 × 1 cm^2^ area of prepared specimens were exposed in acid and rest area was masked with 3 M polyester tape, while 1 × 1 cm^2^ Cu substrates were used for surface morphology analysis.

### Film formation and transfer over copper substrate

A glass petri dish (ϕ = 8 cm) was filled with distilled water up to ¾ of its height and upper water surface was cleaned multiple times with small strips of lint free tissue, to avoid any possible contaminations. One drop (10 µL) of SQR solution in chloroform (10 mg mL^−1^) was released over water surface in the center and a circular floating film of blue color was formed at air-water interface within seconds. Then the floating film was carefully lifted on Cu strip (6 × 1 cm^2^) and washed gently with a stream of distilled water followed by vacuum drying at 50 °C for 1 h. Similarly, multiple layers (up to 4 layers) of SQR film were transferred over Cu substrate and investigated to collect details about layer dependent behavior of Cu corrosion prevention. A schematic presentation showing the real time images during the film formation is given in Fig. 2.

### Preparation of the specimen for spectroscopic, XRD and surface analysis

Surface morphology of SQR deposited Cu substrates (1 × 1 cm^2^) were investigated on HRSEM. Along with the fresh film samples, acid treated samples (0.1 M HCl, 3 h) were also examined to observe the change in surface morphology. For studying the optical and structural properties of SQR, 10 µL of SQR was coated on glass substrates (1 × 1 cm^2^) by FFTMand used to obtain photoluminescence (PL) spectra, UV-vis spectra and XRD patterns.

### Characterization and corrosion testing

Optical properties of SQR were investigated by UV-Vis spectrophotometer of Shimadzu (UV-2600) and fluorescence spectrophotometer of Hitachi (F-4600). TEM micrographs were recorded on FEI-TECHNAI G^2^ 20 TWIN microscope (with accelerating voltage of 200 kV). Film samples for TEM were directly lifted on carbon coated Cu grids. Surface images were recorded on HRSEM, FEI Netherlands (model: Nova Nanosem 450). SSEIS and Tafel polarization curves study was performed on an electrochemical workstation of CH instruments (CHI7041C). A traditional electrochemical set up of three electrodes was used to perform the experiments: working, with and without SQR deposited Cu substrate as electrode; counter, Metrohm tubular Pt electrode; and reference, Metrohm Ag/AgCl tubular electrode. Differential Pulse Voltammetry (DPV) experiments were carried out on PGSTAT302, Metrohm Autolab, Netherlands. Diffraction pattern of SQR was recorded with thin film X-ray diffraction system (Rigaku, Japan) equipped with GIXD.

## Supplementary information

supplementary information

## Data Availability

The data that are analyzed to quote the findings of this paper are available from the corresponding author of the manuscript upon reasonable request.
