# Supplementary material for: Corrosion prevention of commercial alloys by air-water interface grown, edge on oriented, ultrathin squaraine film
Source: Sci Rep. 2019 Sep 17;9:13488. doi: 10.1038/s41598-019-50092-5 (PMC6748964; doi:10.1038/s41598-019-50092-5)
Supplement: Supplementary file 1 — supplementary information [file 41598_2019_50092_MOESM1_ESM.docx]

**Corrosion prevention of commercial alloys by air-water interface grown, edge on oriented, ultrathin squaraine film**

Rajiv Kumar Pandey, Richa Mishra, Gopal Ji^*^ and Rajiv Prakash^#^

School of Materials science and Technology, Indian Institute of Technology (BHU) Varanasi, Uttar Pradesh 221005, India

^*^Corresponding author’s email: [gopal.ji.ji@gmail.com](mailto:gopal.ji.ji@gmail.com). [gopalji.rs.mst11@itbhu.ac.in](mailto:gopalji.rs.mst11@itbhu.ac.in)

^#^Co-corresponding author’s email: rajivprakash12@yahoo.com

**List of Figures and Tables Captions**

**Figure S1.** HRSEM images and corresponding EDAX mappings of pristine copper substrates coated with (a) 0 layer, (b) 1 layer, (c) 2 layers, (d) 3 layers and (e) 4 layers of SQR film.

**Figure S2**. EDAX spectra of the region showed in Figure S1.

**Figure S3.** HRSEM images and corresponding EDAX mappings of corroded copper substrates coated with (a) 0 layer, (b) 1 layer, (c) 2 layers, (d) 3 layers and (e) 4 layers of SQR film.

**Figure S4.** Schematic for showing corrosion inhibiting activity of SQR.

**Figure S5**. Showing standard deviation in the values of (a) I_corr_, (b) R_P_ and (c) R_ct_ and (d) different inhibition efficiencies_._

**Table S1.** EDAX spectra of the region showed in Figure S1.

**Table S2**. Comparison with other film forming techniques.

**
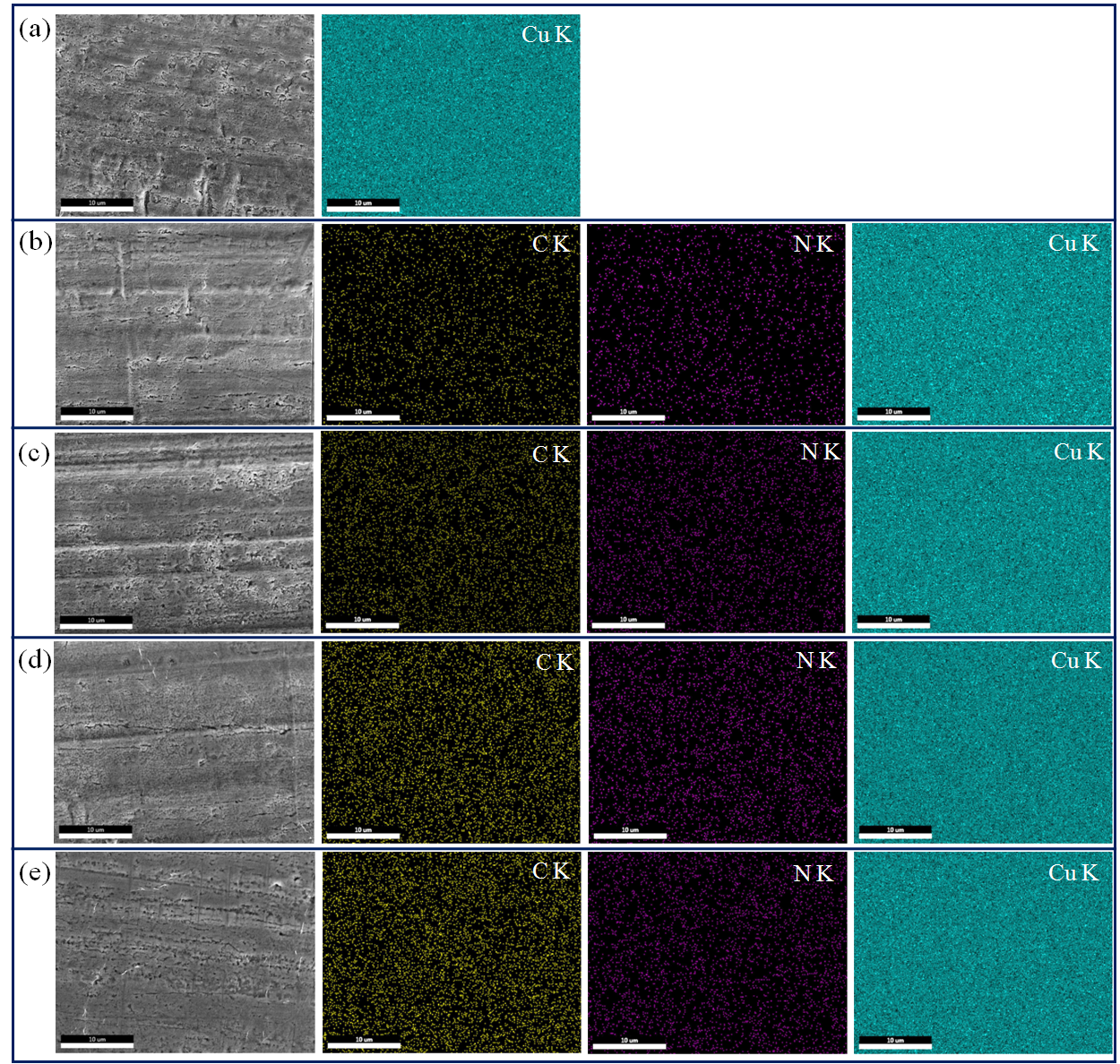
**

**Figure S1**

**
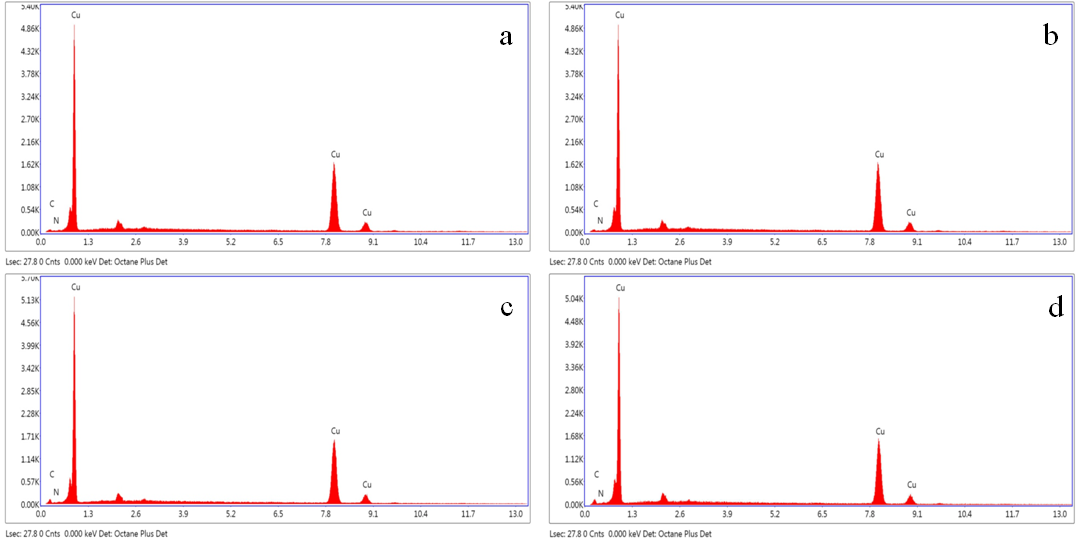
**

**Figure S2**

**
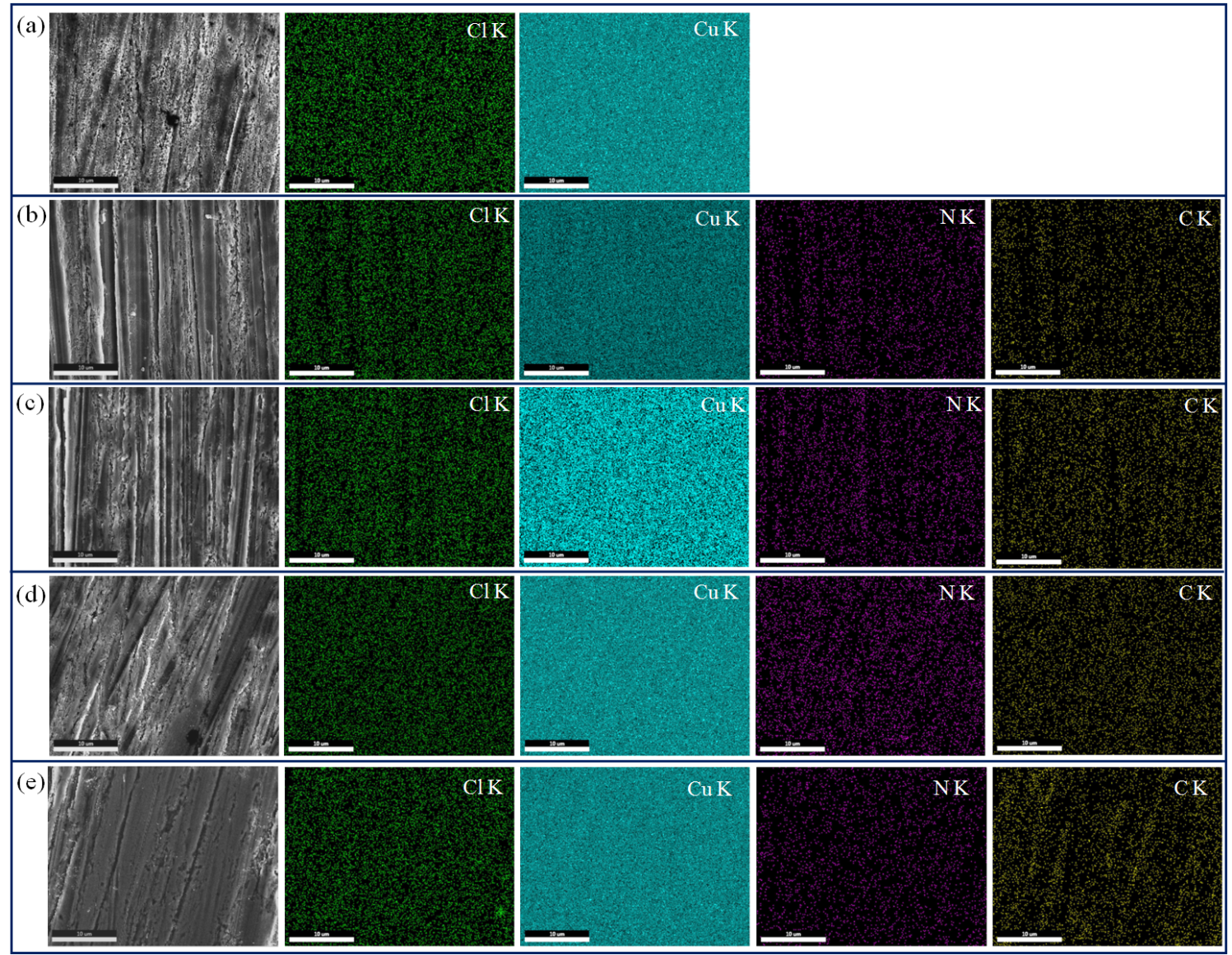
**

**Figure S3**

**
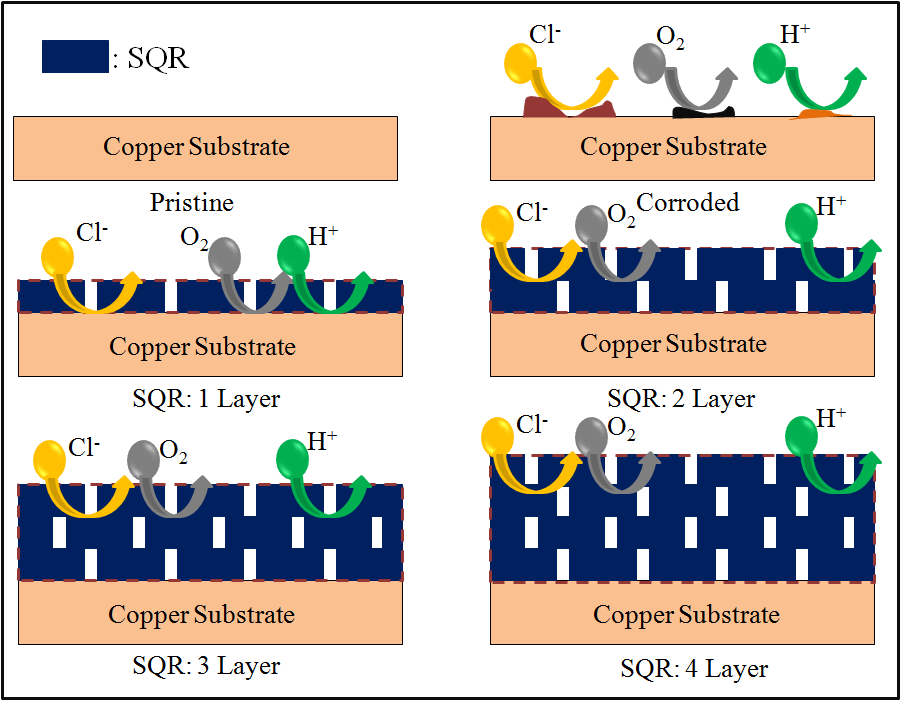
**

**Figure S5**

**Figure S4**


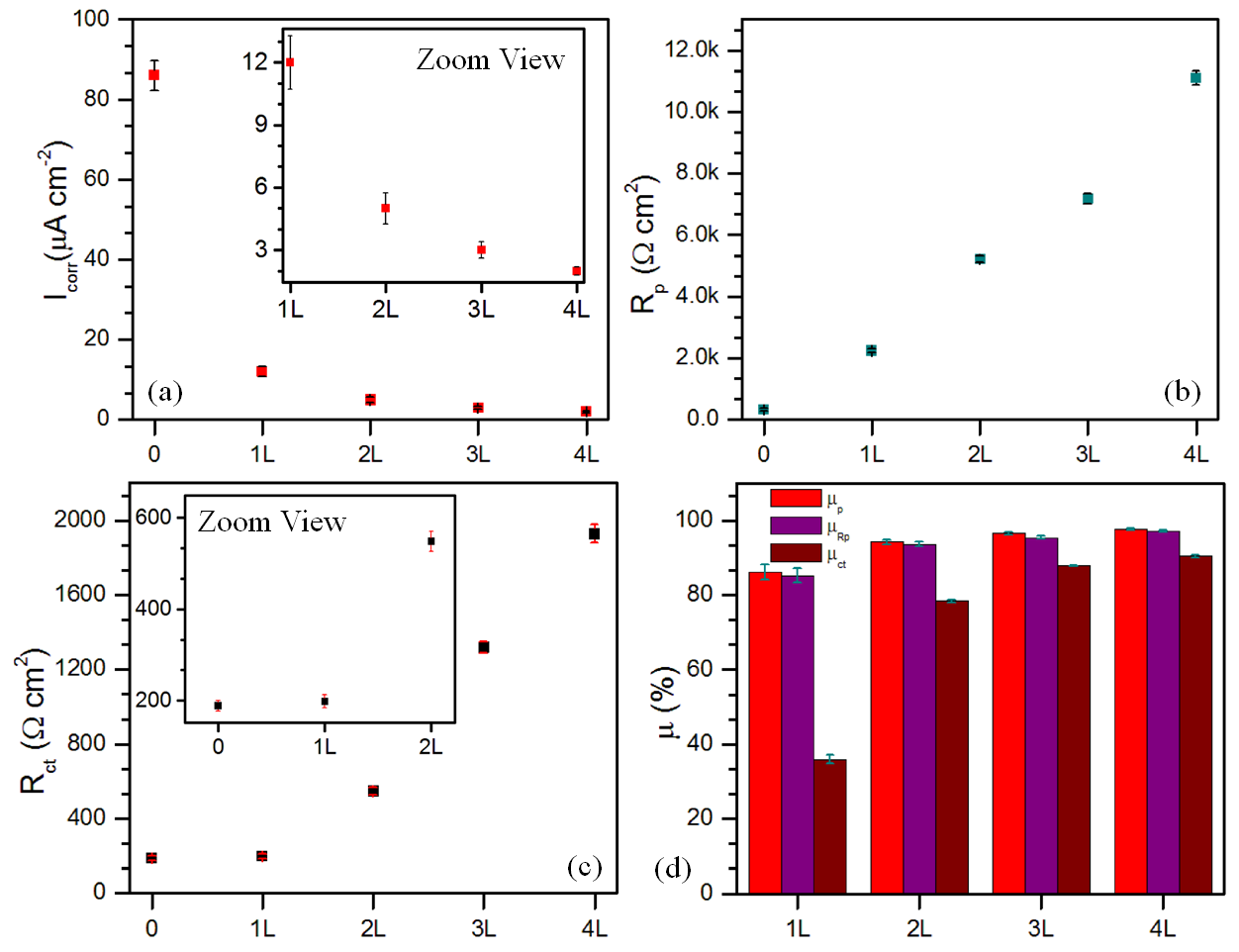


**Figure S5**

**Table S1**

| Samples  Elements | Pristine | | | | | Corroded | | | | |
| --- | --- | --- | --- | --- | --- | --- | --- | --- | --- | --- |
|  | Layer 0 | Layer 1 | Layer 2 | Layer  3 | Layer 4 | Layer 0 | Layer  1 | Layer 2 | Layer  3 | Layer  4 |
| C K | - | 3.92 | 6.51 | 7.90 | 9.47 | - | 0.91 | 2.66 | 4.37 | 8.30 |
| N K | - | 0.02 | 0.17 | 0.18 | 0.24 | - | 0.01 | 0.30 | 0.53 | 1.13 |
| Cu K | 99.96 | 96.06 | 93.32 | 91.92 | 90.29 | 99.65 | 98.83 | 96.83 | 94.94 | 90.46 |
| Cl K | - | - | - | - | - | 0.35 | 0.25 | 0.21 | 0.16 | 0.11 |

**Table S2**

| S. No. | Film Forming Technique | Substrate | Passivating Material | Corrosive Solution | Max Efficiency  (%) | Reference |
| --- | --- | --- | --- | --- | --- | --- |
| 1 | CVD | Copper | Graphene | 0.1 M NaCl | 92 | [1]* |
| 2 | CVD | Nickel | Graphene | 0.5 M HCl | 57 | [2]* |
| 3 | CVD  ALD | Copper  Copper | Graphene | 0.1 m Na_2_So_4_ | 50  99 | [3]* |
| 4 | FFTM | Copper | Squaraine | 0.1 M HCl | 98 | Current Work |

* The references are given below:

[1] Singh Raman, R. K., Chakraborty Banerjee, P., Lobo, D. E., Gullapalli, H., Sumandasa, M., Kumar, A., Choudhary, L., Tkacz, R., Ajayan, P. M., Majumder, M., Protecting Copper from Electrochemical Degradation by Graphene Coating. *Carbon* **2012**, *50*, 4040–4045.

[2] Yu F., Stoot A. C., Boggild P. , Camilli L. , Failure of multi-layer graphene coatings in acidic media *RSC Adv*., **2016**, *6*, 21497–21502 .

[3] Hsieh Y.P., Hofmann M, Chang K.W, Jhu J.G, Li Y.Y, Chen K.Y. , Yang C.C., Chang W.S., Chen L.C., Complete Corrosion Inhibition through Graphene Defect Passivation *ACS Nano* **2014**, 8, 443–448.
